# Non-Destructive Imaging of Defects Using Non-Cooperative 5G Millimeter-Wave Signals

**DOI:** 10.3390/s23146421

**Published:** 2023-07-14

**Authors:** Stavros Vakalis, Jorge R. Colon-Berrios, Daniel Chen, Jeffrey A. Nanzer

**Affiliations:** 1 Department of Electrical Engineering, University of South Florida, Tampa, FL 33620, USA; 2 Department of Electrical and Computer Engineering, Michigan State University, East Lansing, MI 48824, USA

**Keywords:** non-destructive testing, millimeter-wave imaging, 5G signals, joint sensing-communications, passive imaging

## Abstract

Recent developments in fifth-generation (5G) wireless communications networks are creating an increasingly crowded electromagnetic environment at microwave (3–30 GHz) and millimeter-wave (30–300 GHz) frequencies. Radiation at these bands can provide non-destructive testing of defects and shielded structures using non-ionizing signals. In an actual building setting where 5G millimeter-wave communications signals are present, passive imaging of the radiation that is propagating through a wall defect can take place by means of interferometric processing without emitting additional signals in an already-crowded spectrum. We investigate the use of millimeter-wave interferometric imaging of defects in building walls and shielded structures by capturing the transmission of 5G millimeter-wave signals through the defects. We experimentally explore the ability to image defects by capturing the transmission of 38 GHz signals through materials using a 24-element interferometric receiving array.

## 1. Introduction

The ability to perform joint sensing and communication with the same electromagnetic resources is becoming an increasingly important part of future millimeter-wave wireless networks due to the increasingly crowded spectrum, significant power consumption, and limited hardware resources due to the ubiquity of wireless devices around us [1,2]. There are many works that have tried to combine sensing and communications in a joint waveform, but it is challenging because communications and sensing waveforms and hardware are significantly different such that improving the performance of one modality can inhibit the performance of the other [3]. Non-destructive testing waveforms usually exploit amplitude and phase information by sweeping their frequency along a wide synthesized bandwidth [4], while communications signals are instantaneously broadband in order to maximize channel capacity [5].

We present a new approach that can perform non-destructive testing of walls and shielded structures by performing passive millimeter-wave imaging of the ambient electromagnetic signals that get transmitted through the defects. It is achieved with no additional signal emission in an already crowded environment by performing passive interferometric imaging of the millimeter-wave radiation. Our approach can work when the cracks are not visible to the human eye, when for example they are covered by wallpaper, because millimeter-waves can propagate through very thin layers of paper or other materials that are not conductive.

Millimeter-wave imaging is used in a range of applications, including security screening [6,7], non-destructive evaluation [4,8,9], medical imaging [10,11], and remote sensing [12,13]. Signals in the millimeter-wave band of the electromagnetic spectrum with frequencies between 30 and 300 GHz can exhibit fine imaging resolution and very good propagation characteristics through multiple materials including clothing, fog, clouds, and smoke [14,15]. Signals in the millimeter-wave band are non-ionizing, which means that they are safe for use with the general population. However, millimeter-wave radiation cannot easily propagate through cement walls, which makes signals in the millimeter-wave bands a useful tool for inspecting defects and holes in buildings and other structures. Defects and holes can enable the short wavelengths of millimeter-wave radiation to pass through. Millimeter-wave systems are getting increasingly affordable and compact, which makes millimeter-wave technology a very good fit for applications around civilians, such as non-destructive testing, building inspection, and search and rescue.

Interferometric imaging has been used for decades in radio astronomy where significantly sparse antenna arrays capture the signals coming from the stars and other astronomical objects [16,17]. The main motivation was that in order to resolve galactic objects thousands of miles away, very fine angular resolution is essential, which can result in antenna apertures with dimensions in the order of miles or more. Interferometry utilizes widely separated antennas in a sparse formation to generate images with a resolution similar to an actual antenna with the same dimensions. The interferometric image formation process used in these arrays is governed by the van Cittert–Zernike theorem [18,19]. The theorem suggests that the correlations in a sparse antenna array can give rise to the Fourier transform of an incoherent source. The Fourier transform of the incoherent source intensity is usually referred to as the two-dimensional complex scene visibility V(u,v), where u,v are the two spatial frequency dimensions.

Unfortunately, image reconstruction utilizing the van Cittert–Zernike theorem can only take place when the scene is spatially incoherent. Incoherence in space is not always satisfied; an example of this is seen in active radars and typical non-destructive evaluation systems that utilize coherent signal transmission [20,21]. Spatial incoherence translates to sufficient independence between the radiation emitted or scattered by every spatial point in the scene. Therefore, coherent radar cannot utilize sparse interferometric processing, and many times, this can lead to large, heavy, and expensive apertures. On the other hand, thermal radiation coming from astronomical objects satisfies the incoherence requirements due to the randomness of thermal motions of particles. This is the reason why interferometric antenna arrays are an excellent fit for radio astronomy applications. More recently, interferometric imaging systems have been used for security screening applications capturing the thermal radiation from humans [22,23]. The challenge with passive millimeter-wave imaging systems is that electromagnetic signals emanating from thermal sources have extremely low power at millimeter-wave frequencies. The sensitivity ΔT of a radiometric receiver is related to the square root of the system bandwidth *B* and integration time τ by [14]

(1) $$\Delta T=C\frac{T_{sys}}{\sqrt{B\tau }}$$

where $T_{sys}$ is the system noise temperature, and *C* is a constant that depends on the receiver architecture, amplifier noise factor, and amplifier gain. This means that in order for a passive interferometric receiver to have the sensitivity necessary to detect low-power thermally generated signals, they require amplifiers with very high gain, low noise factor, wide bandwidth, and long observation times. This can increase the system cost tremendously and make real-time operation challenging.

We recently presented a new incoherent imaging technique that combats the very high sensitivity requirements by utilizing active incoherent noise transmission at a sparse set of locations [24,25]. This allows the use of commercial low-cost hardware in sparse array formations [26,27], avoids the high-cost counterparts used in synthetic aperture interferometric radiometers, and also can help with achieving image reconstructions at very high frame rates [28]. We have also demonstrated the use of WiFi signals incident on a scene instead of noise transmission, using quadrature amplitude modulated signals with 16 states (16-QAM) [29], thus showing that signals of opportunity can yield sufficient incoherence for image formation. We further demonstrated the use of 5G millimeter-wave frequencies, where we showed imagery using 38 GHz 256-QAM signals [30].

**Figure 1:**
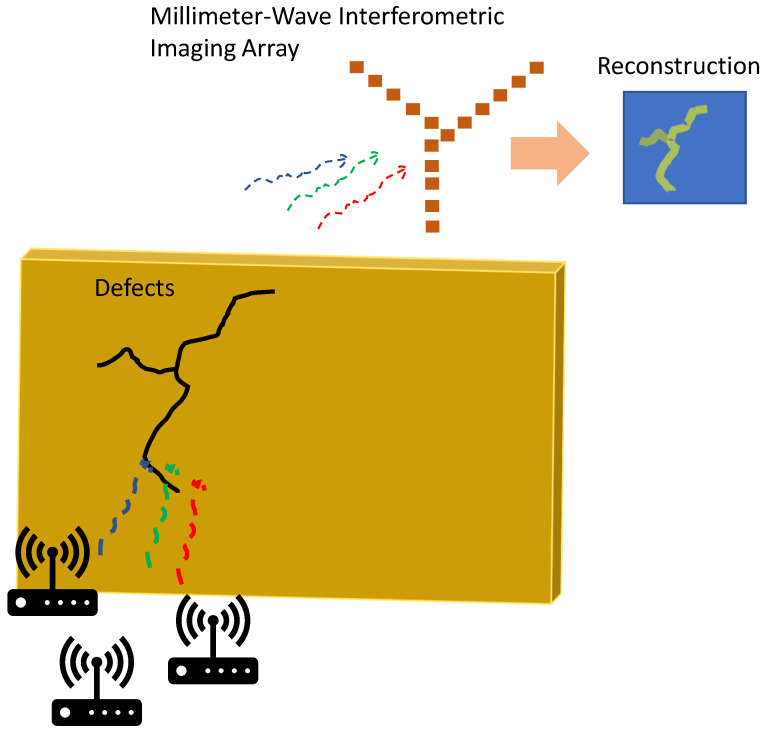
Non-destructive millimeter-wave inspection of defects by capturing transmission of existing millimeter-wave signals through the materials of interest. The signals are captured using a millimeter-wave interferometric antenna array with image reconstruction taking place through Fourier processing.

In this work, we utilize existing wireless millimeter-wave signals in an indoor environment in order to perform imaging of defects and slits as shown in the concept of Figure 1. The existing communication signals will pass through the holes and defects in the wall, and an interferometric antenna array can capture these signals and perform image reconstruction of the transmission. Additionally, unlike most state-of-the-art passive interferometric imaging systems, we do not have to rely on the very low-power thermal signals, because stray communications signals that experience one-way transmission will have orders of magnitude higher power. This makes our approach realizable with commercial components in a low-cost setting. We show experimental measurements of imaging through various defect sizes in a conducting wall using a 24-element 38 GHz imaging array.

### Interferometry Fundamentals

Interferometric processing is usually referred to as correlation processing because the correlations of the electric fields $\langle E_{i}\left(t\right)E_{j}\left(t\right)\rangle$ collected by antenna elements i,j at locations give rise to visibility or spatial Fourier samples given by

(2) $$V_{s}\left(u=\frac{x_{i}-x_{j}}{\lambda },v=\frac{y_{i}-y_{j}}{\lambda }\right)=\langle E_{i}\left(t\right)E_{j}\left(t\right)\rangle$$

where $\left(x_{i},y_{i}\right)$, $\left(x_{j},y_{j}\right)$ are the antenna locations, and λ is the center frequency wavelength.

The total set of cross-correlations between the different antenna locations present in an interferometric aperture is defining the sampling function S(u,v), which can be calculated through

(3) $$S\left(u=\frac{x_{i}-x_{j}}{\lambda },v=\frac{y_{i}-y_{j}}{\lambda }\right)=\sum_{n}^{N}\sum_{m}^{M}\delta \left(u-u_{n}\right)\delta \left(v-v_{m}\right)$$

where u,v are the two spatial frequency dimensions, and N·M is the maximum number of antenna pairs present in the aperture. The sampled visibility $V_{s}\left(u,v\right)$ dictates the amount of information captured through a multiplication sampling function S(u,v) of the array with the scene visibility V(u,v) as in

(4) $$V_{s}\left(u,v\right)=S\left(u,v\right)\cdot V\left(u,v\right).$$

The two-dimensional reconstructed scene intensity $I_{r}$ is the two-dimensional inverse Fourier transform of the scene visibility $V_{s}\left(u,v\right)$, which can be calculated by

(5) $$I_{r}\left(\alpha ,\beta \right)=\sum_{n}^{N}\sum_{m}^{M}V_{s}\left(u_{n},v_{m}\right)e^{-j2\pi (u_{n}\alpha +v_{m}\beta )}$$

where α=sinθcosϕ and β=sinθsinϕ are the direction cosines relative to the *u* and *v* spatial frequency dimensions.

Although interferometric image reconstruction is primarily a Fourier-domain process as the correlations are directly mapped to the scene visibility, the spatial-domain interpretation can be very helpful for understanding spatial characteristics such as resolution and field of view. The spatial-domain impulse response of the imaging system is the point-spread function (PSF) of the array, where

(6) PSF(α,β)=IFT{S(u,v)}.

The PSF usually consists of a synthesized beam in the spatial domain with a number of sidelobes, and is a result of the different baselines included in the array. Because interferometric imaging is incoherent imaging and reconstructs image intensities, we utilize the squared magnitude ${\left|\mathrm{PSF}\right|}^{2}$. The reconstructed scene intensity $I_{r}$ can be written as a convolution between the scene intensity *I* and the PSF in the spatial domain:

(7) $$I_{r}\left(\alpha ,\beta \right)={\left|\mathrm{PSF}\left(\alpha ,\beta \right)\right|}^{2}*I\left(\alpha ,\beta \right)$$

where ∗ indicates convolution in the spatial domain. The reconstructed scene intensity will be shaped by the shape of the PSF. In the ideal case, the PSF will be identical with a delta function, which can be written as PSF(α,β)=δ(α,β), and therefore $I_{r}\left(\alpha ,\beta \right)=I\left(\alpha ,\beta \right)$. Unfortunately, in order for the PSF to be a delta function, the sampling function S(u,v) should extend from −∞ to +∞ in the spatial frequency domain, which translates to an infinite number of antennas that is not realizable in actual systems.

**Figure 2:**
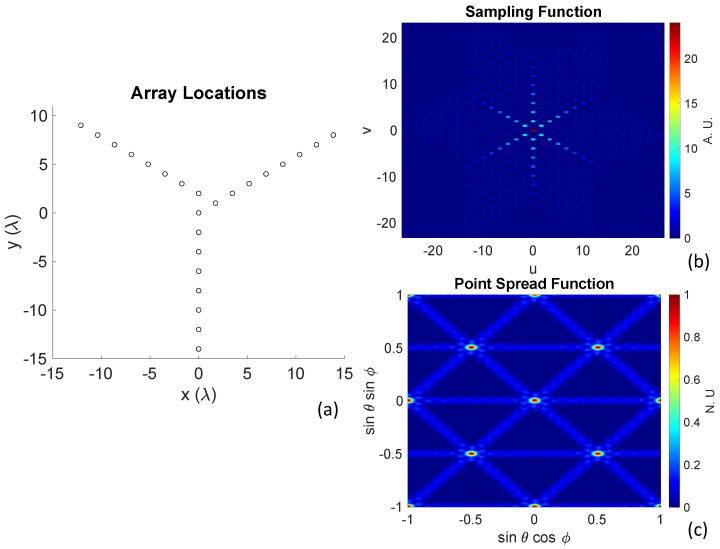
(**a**) Array layout of a 24-element asymmetric Y-shaped array. (**b**) Sampling function S(u,v), which is the result of the cross-correlations between all antenna locations and shows the spatial frequency coverage. (**c**) Point-spread function (PSF), which shows the synthesized beam in the spatial domain. As interferometric imaging is a Fourier imaging method, the PSF is usually calculated as an inverse Fourier transform of S(u,v).

The array layout, sampling function S(u,v), and PSF of a 24-element aperture in asymmetric “Y“ formation can be seen in Figure 2a, Figure 2b, and Figure 2c, respectively. The sampling function S(u,v), which shows the spatial frequency coverage, can be found through an autocorrelation of the antenna array aperture, and the PSF can be calculated through an inverse Fourier transform of S(u,v).

Inter-element antenna spacings affect the unambiguous field of view of an interferometric imager. Ideally, λ/2 will give the full $\left(-\frac{\pi }{2},\frac{\pi }{2}\right)$ field of view, but increasing this spacing will give rise to sidelobes. For an array with grid spacings $d_{x}$ and $d_{y}$ in the horizontal and vertical array axes, the unambiguous field of view can be expressed for the two direction cosines α and β as

(8) $$FOV_{\frac{\alpha }{2},\frac{\beta }{2}}=\frac{\lambda }{2\cdot d_{x,y}}.$$

The spatial resolution of an interferometric imager in the azimuth and elevation planes can be approximated by the half-power beamwidth $\theta_{HPBW}$ of the sinc-squared response from the largest baselines in the horizontal and vertical axes of the array *x* and *y* [31]. $\theta_{HPBW}$ can be found through

(9) $$\Delta \theta_{\alpha ,\beta }\approx \theta_{HPBW}^{\left(\alpha ,\beta \right)}\approx 0.89\frac{\lambda }{D_{x,y}}.$$

## 2. Materials and Methods

**Figure 3:**
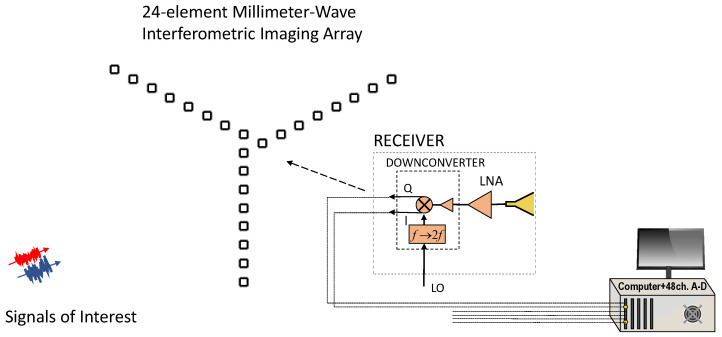
Block diagram of the 24-element millimeter-wave interferometric imaging array, which is capturing the third-party signals of opportunity (shown with blue and red). Quadrature down conversion is implemented using the same 19 GHz local oscillator in all channels. The in-phase and quadrature signals are digitized and processed on the host computer.

**Figure 4:**
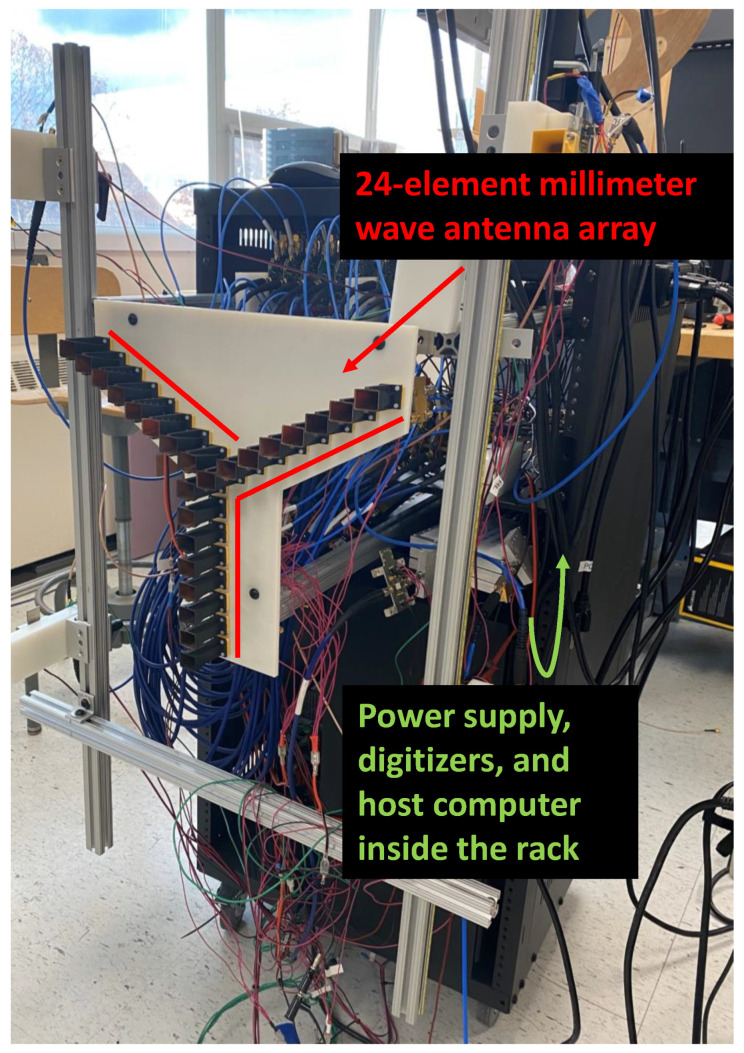
Photograph of the 38 GHz interferometric imaging array setup. The 24-element millimeter-wave antenna array is shown with red.

The interferometric antenna array used in this work had an asymmetric “Y”-shaped formation with 24 elements in 3λ increments. A block diagram of the architecture can be seen in Figure 3. The 24 receivers employed 3D-printed 15-dBi standard-gain horn antennas. Design of the antennas took place using ANSYS High-Frequency Structure Simulator (HFSS). The antennas were printed using VeroWhitePlus material on a Stratasys Objet Connex 350 Multi Material 3-D Printing System. In order to metallize the antennas, the structure was first sputtered with 60 nm titanium for adhesion between copper and printed structure and afterwards by 500 nm copper. Finally, the structure was electroplated with copper to achieve a thickness of 6 μm. A photograph of the antenna array showing the 24 horn antennas can be seen in Figure 4. The inter-element spacing between the antennas was 3λ, which means that the unambiguous field of view of the imager can be found to be equal with $22^{\circ }$ and $38^{\circ }$ in the azimuth and elevations planes, respectively, using (8). The spatial resolution of the imager was $1.3^{\circ }$ and $1.44^{\circ }$ in the azimuth and elevation planes, respectively, based on (9).

**Figure 5:**
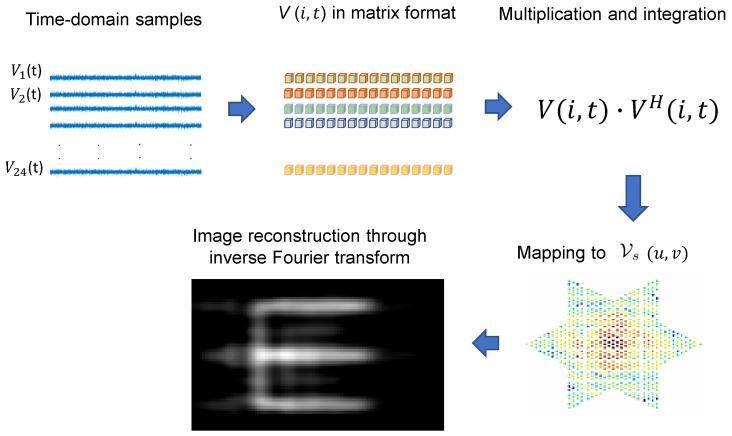
Summary of the digital image reconstruction implementation. The signals of opportunity that are getting transmitted through the defects are captured in time domain, and the algorithm digitally reconstructs the defect shape, shown with the “E”-shaped target.

The 38 GHz signals coming out of the 24 receive antennas were amplified using 23 dB gain analog devices (ADI) HMC1040LP3CE low-noise amplifiers and then quadrature downconverted using ADI HMC6789BLC5A downconverters with integrated local oscillator (LO) frequency doublers fed by a 19 GHz LO. Three 16-channel ATS9416 14-bit, 100 MS/s, AlazarTech waveform digitizers installed on a computer were utilized for the data acquisition of the 48 in-phase and quadrature channels from the 24 antennas. The three digitizers were frequency lockedm and triggering was taking place in the time domain using a common 1 kHz signal in order to eliminate frequency differences or timing jitter between the 48 baseband channels (24 complex signals). The signal processing and image reconstruction took place in real time using MATLAB on the host computer. An overview of the image reconstruction algorithm can be seen in Figure 5. The transmitted signals of opportunity are sampled at the 24 receive antenna locations, and they are quadrature downconverted. The signals V(t) are complex, and both their in-phase (I) and quadrature (Q) components are captured simultaneously with 48 parallel digitizer channels. In order for the cross-correlations to take place between all the antenna pairs present in the array, we need to take the dot product between every two antenna elements. In order to achieve that, we multiply V(i,t), which is the complex response of the *i*th element, with its conjugate transpose. By doing that, every row of the matrix V(i,t) is multiplied with every column of $V^{H}\left(j,t\right)$, which is the conjugate response of the *j*th element, and then summed (integrated). After the multiplication between the two matrices takes place, the cross-correlations are translated to visibility samples $V_{s}$ based on the antenna pairs generating the samples, and finally, the reconstructed scene intensity is computed through an inverse Fourier transform (IFT) [28]. The reconstructed image will be blurred due to the finite beamwidth of the PSF and its sidelobes, and will also have noise.

A regularizer function is frequently used to mitigate the noise present in the image and compensate for the lost information during the blurring. The regularizer function used here for non-destructive imaging, which captures piecewise constant intensities as a function of millimeter-wave signal transmission, is the total variation TV(**x**) of the image **x**, which can be written as

(10) $$\mathrm{TV}\left(x\right)=\sum_{i}\sqrt{{\left(\Delta_{i}^{H}x\right)}^{2}+{\left(\Delta_{i}^{V}x\right)}^{2}}$$

where $\Delta_{i}^{H}$ and $\Delta_{i}^{V}$ are the finite differences operator along the horizontal and vertical dimensions of **x** [32]. In this way, we can deblur the image while also achieving denoising at the same time.

## 3. Results

**Figure 6:**
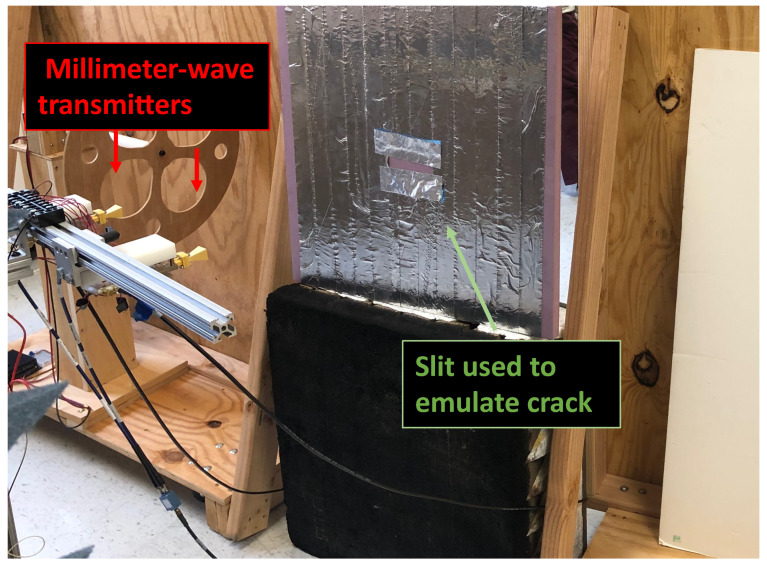
Experimental setup that shows two millimeter-wave transmitters, and the aluminum-tape-covered target with a slit opening that is used to emulate defects.

**Figure 7:**
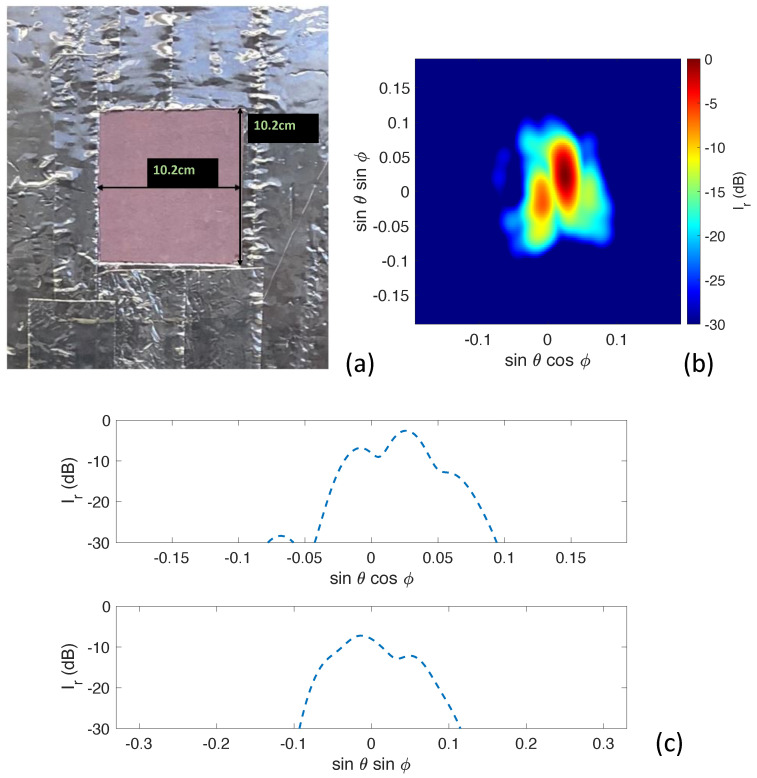
(**a**) Foam board covered in aluminum tape with a square opening used as the transmission target. (**b**) Experimental image reconstruction of the target square opening. (**c**) One-dimensional slices along sinθsinϕ = 0 (**top**) and sinθcosϕ = 0 (**bottom**).

Experimental measurements took place using two 38 GHz transmitters that utilized ADI HMC6787A upconverters and ADI HMC7229 power amplifiers. The setup is shown in Figure 6. The interferometric imaging system is at the other side of the wall within 1.80 m from the target and captures the transmitted signals. The transmitter antennas are located at 0.61 m from the wall, transmitting a single tone signal at 38 GHz. The first target we inspected was a foam board covered in aluminum foil with a square aperture opening, as shown in Figure 7a. The aluminum foil allows only transmission through the aperture, mimicking, for example, the absorption of concrete in a wall. We note that the presented scenario does not exactly match the propagation losses in a wall, but many wall materials at millimeter-wave frequencies will exhibit high reflectivity and very low transmission, which is similar to our experimental setup. The foam board had dimensions of 61 by 61 cm. The square had dimensions of 10.2 by 10.2 cm.

The reconstruction of the transmitted signals can be seen in Figure 7b which verifies the idea of Fourier-domain image reconstruction of signals transmitted through a wall defect for non-destructive imaging. We also took two one-dimensional slices along sinθsinϕ = 0 and sinθcosϕ = 0, which can be seen in top and bottom of Figure 7c, respectively. Both one-dimensional cuts show good dynamic range for further evaluation and classification of the square.

**Figure 8:**
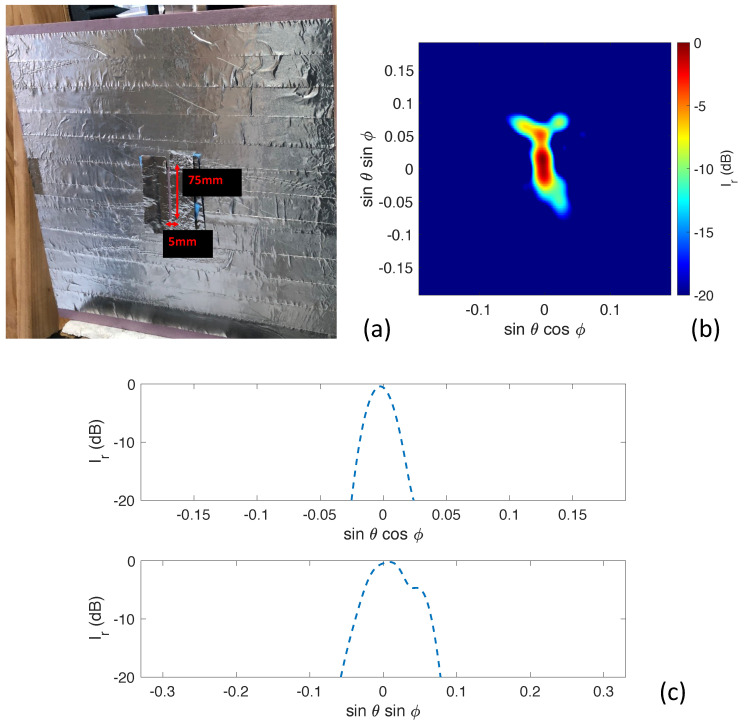
(**a**) Foam board covered in aluminum foil with a slit used as the transmission target. (**b**) Experimental image reconstruction of the slit. (**c**) One-dimensional slices along sinθsinϕ = 0 (**top**) and sinθcosϕ = 0 (**bottom**).

The next target we examined was a foam board with the same dimensions, but which had a much smaller defect represented by a slit opening of 5 mm by 75 mm simulating a wall defect, as shown in Figure 8a. This represents a scenario that mimics a small wall crack. The reconstructed millimeter-wave image of the slit can be seen in Figure 8b. The experimental proof-of-concept shows good image reconstruction capabilities. The opening appears a little bit larger in our reconstructed image because of diffracted electromagnetic fields. The two one-dimensional slices along sinθsinϕ = 0 and sinθcosϕ = 0 can be seen in top and bottom of Figure 8c, respectively. Both the azimuth and elevation cuts show good dynamic range, which can be used for further inspection and classification.

## 4. Conclusions

In this article, we presented a new method of detecting and imaging defects and openings in walls utilizing existing 5G wireless network infrastructure and a receive interferometric array. While most other microwave imaging methods utilize reflections from transmit and receive apertures, we utilize only receivers and the existing electromagnetic radiation in the environment which suggests lower power and complexity. The proof-of-concept 5G experimental images of slits show promise for future non-destructive inspections using sparse antenna arrays. We showed experimental measurements of non-destructive imaging at 38 GHz of square openings and slits using non-cooperative signals from third party transmitters. Both reconstructions show good dynamic range, which can lead to further analysis and classification. This work is a first step towards contactless and non-ionizing inspection of buildings and other structures using existing communications signals or other incoherent “beacon” signal architectures. This means that the operating frequency and bandwidth will not be limited by the 5G spectrum, as there is the potential for utilizing incoherent transmit architectures. Future work will include experimental results through different wall materials and thicknesses and discuss imaging performance.

## Data Availability

Not applicable.

## Abbreviations

The following abbreviations are used in this manuscript:

HPBW	Half-Power Beamwidth
FOV	Field of View
LO	Local Oscillator
PSF	Point-Spread Function
IFT	Inverse Fourier Transform
QAM	Quadrature Amplitude Modulation
TV	Total Variation
